# Clinical characteristics of human fascioliasis in Egypt

**DOI:** 10.1038/s41598-023-42957-7

**Published:** 2023-09-27

**Authors:** Nagat Ibrahim, Ekram M. Abdel Khalek, Nahed A. Makhlouf, Muhammad Abdel-Gawad, Mohamed Mekky, Haidi Karam-Allah Ramadan, Ahmed Abu-Elfatth, Naglaa Abd El-Latif, Marwa Khalaf Hassan, Rabab Eldeeb, Mohamed Abdelmalek, Sherief Abd-elsalam, Hanaa Attia, Ahmed Qasem Mohammed, Hani Aboalam, Mahmoud Farouk, Mohamed Alboraie

**Affiliations:** 1 Department of Gastroenterology, Hepatology, and Infectious Diseases, Assiut Liver Center, Ministry of Health, Assiut, Egypt; 2 Public Health and Community Medicine, Assiut University, Assiut, Egypt; 3 Tropical Medicine and Gastroenterology Department, Faculty of Medicine, Assiut University, Assiut, Egypt; 4 Hepatology, Gastroenterology, and Infectious Diseases Department, Al-Azhar University, Assiut, 71631 Egypt; 5 Parasitology Department, Medical Research Institute, Alexandria University, Alexandria, Egypt; 6 Tropical Medicine and Gastroenterology, Assiut Liver Center, Ministry of Health, Assiut, Egypt; 7 Tropical Medicine, Alexandria University, Alexandria, Egypt; 8 Tropical Medicine and Infectious Diseases, Tanta University, Tanta, Egypt; 9 General Manager of Endemic Diseases Control, Ministry of Health, Behera, Egypt; 10 Department of Tropical Medicine and Gastroenterology, Luxor University, Luxor, Egypt; 11 Department of Internal Medicine, Al-Azhar University, Cairo, Egypt

**Keywords:** Zoology, Diseases, Gastroenterology

## Abstract

There is a lack of epidemiological data on fascioliasis in Egypt regarding disease characteristics and treatment outcomes across different governorates. We aimed to identify the demographic, epidemiologic, clinical, laboratory, and radiological characteristics and treatment outcomes of patients diagnosed with fascioliasis in Egypt. Data on human fascioliasis were collected retrospectively from patients’ medical records in the period between January 2018 and January 2020. The study included 261 patients. More than 40% of enrolled patients were in the age group of 21–40 years old. Geographically, 247 (94.6%) were from Assiut Governorate with 69.3% were from rural areas. The most frequent symptoms were right upper quadrant pain (96.9%), and fever (80.1%). Eosinophilia was found in 250 cases (95.8%). Hepatic focal lesions were detected in 131 (50.2%); out of them 64/131 (48.9%) had a single lesion. All patients received a single dose of 10 mg/kg of triclabendazole, 79.7% responded well to a single dose, while in 20.3% a second ± a third dose of treatment was requested. After therapy, there was a reduction in leucocytes, *Fasciola* antibodies titer, eosinophilic count, bilirubin, and liver enzymes with an increase in hemoglobin level. According to our findings, a high index of suspicion should be raised in cases with fever, right upper abdominal pain, and peripheral eosinophilia, and further imaging workup is mandated to detect hepatic focal lesions. Prompt treatment by triclabendazole can serve as a standard-of-care regimen even for suspected cases.

## Introduction

More than eighty countries throughout the globe have reported cases of fascioliasis, a zoonotic infection spreads by eating contaminated food^1^. This infection is challenging to contain due to its complex epidemiology. Two species of liver flukes, *Fasciola hepatica*, and *Fasciola gigantica* are responsible for this parasitic disease. *Fasciola hepatica* are found all over the world since their snail vectors are present everywhere, but *Fasciola gigantica* is limited to Africa and Asia^2^. Significant economic losses and expenditures are caused by fascioliasis infestation in cattle, including impaired fertility, decreased meat, milk, and wool production, cost of anthelmintic medications, reduced weight gain, and loss from death^3^. Around 17 million individuals are infected with *Fasciola* globally^4–7^, and cases of high pathogenicity, such as neurological and ophthalmological affections, result in long-lasting consequences and even death^5^. It is found in Oceania, Asia, Africa, the Middle East, Europe, the Caribbean, and parts of Latin America. In some areas where animal fascioliasis is found, human cases are uncommon or sporadic. In other areas, human fascioliasis is very common or hyperendemic^4,7^.

Additionally, *Fasciola* infection prevalence varies greatly among regions^8–10^. Rates of infection ranged from 0 to 68% among 2700 participants investigated by Esteban et al. in 24 communities distributed throughout a narrow region between La Paz and Lake Titicaca in the Bolivian Altiplano^11^. Additionally, Cabada et al. evaluated 2500 children in 26 neighboring towns in the Anta province in Peru and found infection rates ranging from 0 to 20%^12^. Fascioliasis has been reported to be reemerging and emerging in various African, Asian, and Middle Eastern nations, with Iran, Turkey, Egypt, and Vietnam being the main endemic countries^13–16^. *F*asciola eggs have been detected in a mummy, confirming that human fascioliasis has existed since pharaonic times^17^. Human fascioliasis has been identified in almost all Delta governorates^16,18^. In the Behera Governorate, coprological studies have shown a very high prevalence ranging from 5.2 to 19.0%^19^. Egypt had a very high prevalence^20–22^, which suggests that earlier WHO reports may underestimate the real situation^19^. Humans are infected by many different sources, which vary according to countries, diet and traditions. Sources mainly include several vegetables, drinking of natural freshwater or combinations of both, transporting the infective stage of metacercaria^23^.

Effective preventative actions may be implemented with an understanding of the disease’s epidemiology and its determinants. This study was designed to identify the demographic, epidemiological, clinical, laboratory, radiological characteristics and treatment outcomes of patients diagnosed with human fascioliasis in two governorates of Egypt.

## Materials and methods

### Study design

The current study was a retrospective study.

### Study sites

Endemic diseases clinic in Assiut Governorate and Endemic Diseases Departments at the Directorate of Health Affairs in Al-Behera, Egypt.

### Study population

The study retrospectively included the medical records of patients diagnosed with fascioliasis from January 2018 to January 2020 as during this duration an outbreak of *Fasciola* infection occurred at Manfalout District in Assiut Governorate. The records of patients who didn’t meet the criteria of diagnosis of fascioliasis (peripheral eosinophilia, positive stool analysis for *Fasciola* ova (Kato Katz test), and/or positive anti-*Fasciola* antibody) were excluded from the study. Indirect Hemoagglutination assay was used for detection of *F*asciola Antibody with a titer of 160 or more was considered to be positive.

### Study tool and data collection

A structured data sheet was designed and included demographic data, baseline clinical data, laboratory and radiological investigations, treatment outcome. Patients were followed up for 3 months after treatment by clinical examination, laboratory, and radiological investigations. The Kato Katz test was used for microscopic stool examination of eggs detection.

Data was collected from 370 medical records; 315 records from Assiut and 55 from Al- Behera were included, with the exclusion of 109 records because of a deficiency of some relevant data (Supplementary Table 1).

### Ethics considerations

Obtaining the approval of the proposal from the Ethics Committee at the Central Directorate of Research and Health Development and review in the Ministry of Health and Population study (Approval number: 5-2021/12 on 10 March 2021). Official permission was obtained to access data from the Endemic Diseases Department at the Directorates of Health Affairs in Assiut and Al-Behera Governorates. Privacy and confidentiality of all data were assured as data sheets were coded with numbers to maintain anonymity. The study was conducted in accordance with the Helsinki Declaration’s ethical guidelines. As the study is retrospective, patients' consent was waived by the Ethics Committee at the Central Directorate of Research and Health Development and review in the Egyptian Ministry of Health and Population study.

### Data management and analysis

Data was collected and reviewed carefully to ensure data quality. Data entry, cleaning recording, and analysis were done using SPSS software version 26 (IBM SPSS Inc., Chicago, US) for windows 10. Descriptive statistics were calculated as the mean and standard deviation (SD) for normally distributed quantitative variables and as a median and interquartile range for non-parametric quantitative variables and as frequency and percentages for categorical variables. Paired t-test was used as the test of significance for normally distributed quantitative variables and Wilcoxon signed-rank test was used for comparing the difference between variables before and after treatment. The correlation between variables was tested using the spearman correlation**.** Statistical significance was considered when p-value was ≤ 0.05 for all statistical tests.

## Results

**Figure 1:**
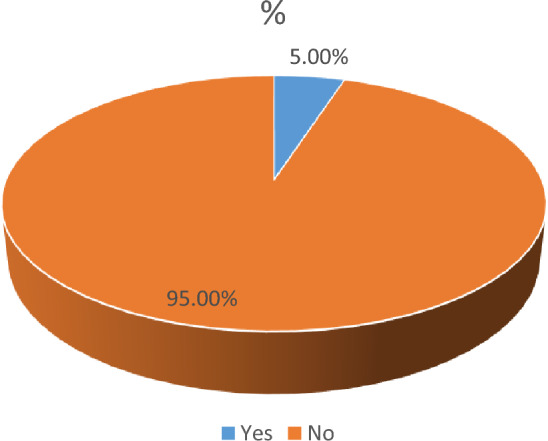
Presence of *Fasciola* egg in the examined stool before treatment.

A total of 261 patient medical records were included, the mean age of the enrolled patients was 33.3 ± 17.9 years with a range between 5 and 75 years. More than half of the patients (55.2%) were females. The vast majority (94.6%) of cases were from Assiut Governorate. Rural residents represented 69.3% of the patients included. Regarding occupation, 37.2% were housewives, 30.7% were students, and 9.6% were farmers (Table 1). The majority of the patients (85.8%) came from Manfalout District, Assiut Governorate (Supplementary Fig. 1). Table 2 shows the baseline clinical manifestations of the included patients. The median duration of symptoms was 30 days with a range between 5 and 160 days. The most frequent symptoms were right upper quadrant pain (96.9%), fever (80.1%), and nausea (55.2%). Jaundice and dark urine were reported in 18 patients’ records (6.9%). As shown in Table 3, abdominal computed tomography (CT) was performed in 32 (12.3%). But abdominal ultrasound was done for all patients. It was found that 23.4% of the patients had hepatomegaly, 24.5% had a single hepatic focal lesion and 25.7% had multiple lesions. The hepatic lesion was commonly observed in the right lobe (61.1%). Before treatment, 16 patients (6.1%) viable worms could be detected in the biliary system and were extracted with ERCP. Regarding the baseline laboratory data, *Fasciola* egg was detected in 5% of the examined stool as most of our patients were in the acute phase and some patients did not provide a stool specimen for analysis (children and a few adults), (Fig. 1). Unfortunately, eggs were measured in no case, so that a specific diagnosis whether by *F*. *gigantica* or *F*. *hepatica* could not be made”.

**Table 1:** Demographic data of the included patients.

	No. (261)	%
Sex		
Male	117	44.8%
Female	144	55.2%
Age: (years)		
Mean ± SD	33.33 ± 17.93	
Median (range)	33.0 (5.0–75.0)	
Governorate		
Assiut governorate	247	94.6%
Beheira governorate	14	5.4%
Residence		
Rural	181	69.3%
Urban	80	30.7%
Marital status		
Married	153	58.6%
Unmarried	108	41.4%
Occupation		
Housewife	97	37.2%
Farmer	25	9.6%
Employee	13	5.0%
Worker	41	15.7%
Student	80	30.7%
Not working	5	1.9%
Smoking		
Smoker	22	8.4%
Non-smoker	239	91.6%

**Table 2:** Clinical manifestations of the included patients.

Variable	No. (261)	%
Right upper abdominal pain	253	96.9
Fever	209	80.1
Nausea	144	55.2
Fatigue	77	29.5
Tender hepatomegaly	61	23.4
Vomiting	58	22.2
Itching	38	14.6
Pallor	28	10.7
Dark urine (bilirubinuria)	18	6.9
Yellow sclera	18	6.9
Weight loss	14	5.4
Epigastric pain	8	3.1
Anorexia	6	2.3
Diarrhea	6	2.3
Rash	2	0.8
Bleeding tendency	1	0.4
Duration of symptoms (in days)		
Median (range)	30 (5–160)

**Table 3:** Imaging findings in the included patients by ultrasound or computed tomography.

Variable	No. (261)	%
Computed tomography (CT)		
Done	32	12.3
Not done	229	87.7
Hepatomegaly		
Yes	61	23.4
No	200	76.6
Number of hepatic focal lesion (HFL)		
No	130	49.8
Single	64	24.5
Multiple	67	25.7
Site of HFL: (n = 131)		
Right lobe	80	61.1
Left lobe	26	19.8
Both lobes	25	19.1
Size of largest HFL (in cm)		
Mean ± SD	3.37 ± 1.13	
Median (range)	3.0 (1.3–7.0)	
Splenomegaly	21	8.0
Ascites	15	5.7
Abdominal lymphadenopathy	32	12.3
Viable worms in biliary tree detected by ERCP intervention	16	6.1

*ERCP* endoscopic retrograde cholangiopancreatography.

As shown in Table 4, at the baseline there was a marked increase in *Fasciola* antibody titer 640 (80–1280), eosinophils 3.6 (0–32.9), leucocytes 12 (2.3–45), platelets 304 (110–888), total bilirubin 2.6 (0.3–11), alanine transaminase 56 (14–311), and aspartate transaminase 59 (19.5–287) followed by a significant reduction in these parameters after therapy. On the other hand, there was no significant difference in hemoglobin level (p = 0.064) and albumin (p = 0.637).

**Table 4:** Laboratory investigations of the included patients.

Variable	Baseline median (IQR)	Follow-up median (IQR)	P-value
*Fasciola* antibody titer	640 (80–1280)	160.0 (40.0–1280.0)	0.000*
White blood cells (10^9^/L)	12 (2.3–45)	8 (3–23)	0.000*
Hemoglobin gm/dl (mean ± SD)	12.10 ± 1.55	12.20 ± 1.34	0.064
Platelets (10^9^/L)	304 (110–888)	295(124–633)	0.018*
Eosinophil count (10^9^/L)	3.6 (0–32.9)	0.7 (0–10.1)	0.000*
Eosinophil percent	34 (1.0–85)	9(1.0–53)	0.000*
Total bilirubin (mg/dl)	2.6 (0.3–11)	1 (0.4–2.1)	0.000*
Direct bilirubin (mg/dl)	1.6 (0.1–9)	0.4 (0.1–1.3)	0.000*
ALT (U/L)	56 (14–311)	32 (15–208)	0.000*
AST (U/L)	59 (19.5–287)	33 (17–101)	0.000*
Albumin (gm/dl) (mean ± SD)	4.02 ± 0.45	4.01 ± 0.36	0.637
Alkaline phosphatase (U/L)	327 (67 -923)	231.5 (45 -431)	0.000*

The asterisk means statistically significant. White blood cells (4–10), Hemoglobin (11.7–15.5), Platelets (150- 440), Eosinophil count (0–0.8), Eosinophil percent (0–3), *F*asciola Antibody titer (negative: Less than 160, positive: 160 or more), Total Bilirubin (0–1.2), ALT (0–41), AST (0 -40), Serum Albumin (3.5–5.2), Alkaline phosphatase (44–147).

*ALT* alanine transaminase, *AST* aspartate transaminase.

**Figure 2:**
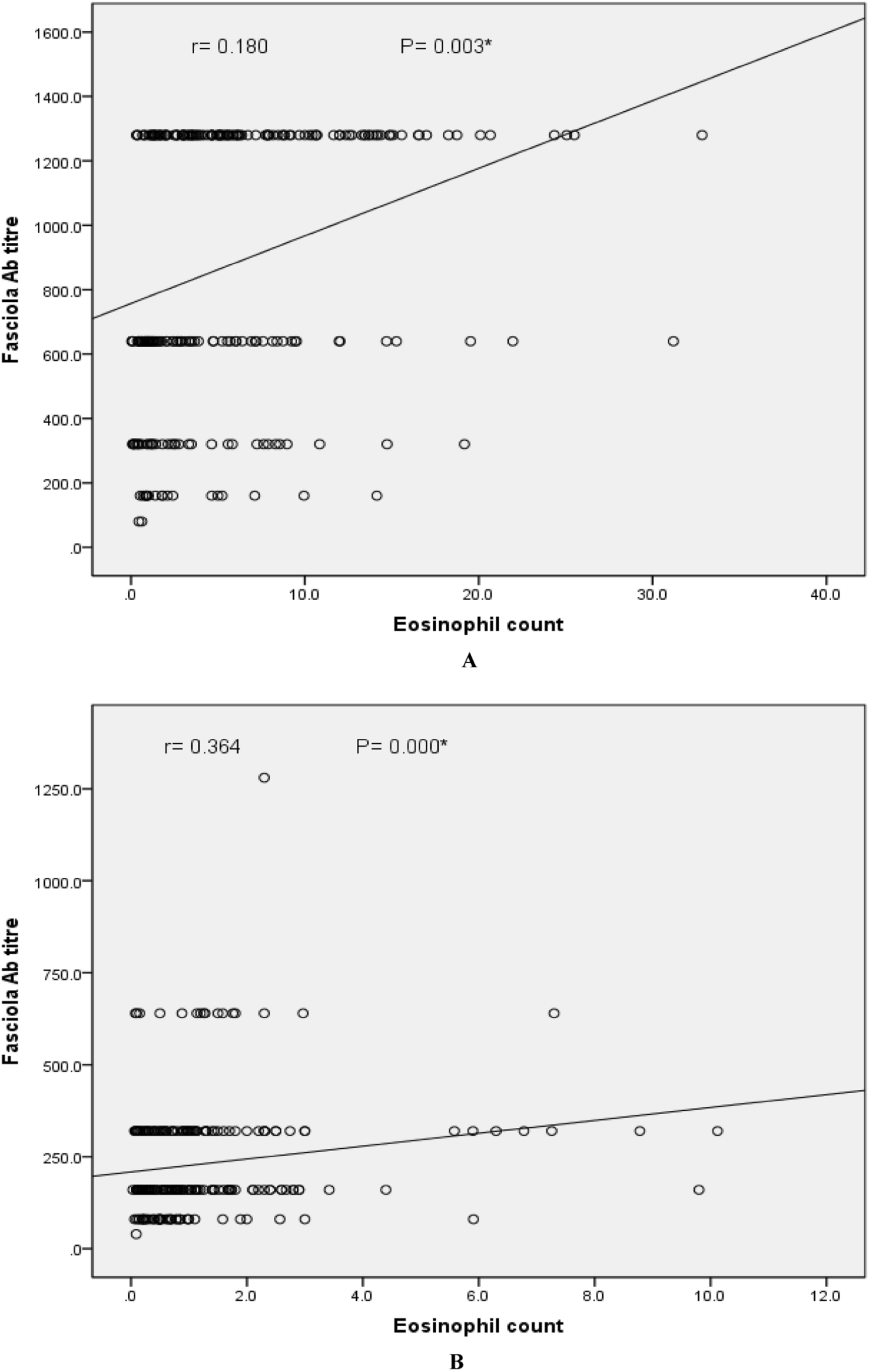
(**A**) Correlation between Eosinophil count and *Fasciola* antibody titer before treatment. (**B**) Correlation between Eosinophil count and *Fasciola* antibody titre after treatment.

Table 5 shows the treatment of the included patients and its outcome. For all patients, a single dose of 10 mg/kg of triclabendazole was described according to the treatment protocol of the Egyptian Ministry of Health and Population during the study period. The response was reported in 79.7% to a single dose, while in 20.3% a second ± a third dose of treatment was requested. Patients were followed up for 3 months following treatment by clinical evaluation, complete blood counts, *F*a*sciola* antibody, stool examination for *Fasciola* eggs and abdominal ultrasound. Clinical improvement was reported in 84.7% while 40 patients suffered from the persistence of abdominal pain (95.0%), nausea/vomiting (40.0%), fatigue (15.0%), and itching (2.5%). *Fasciola* egg in stool was detected in only three patients after therapy. As shown in Fig. 2A, there was a statistically significant positive weak correlation (r = 0.2) between the eosinophil count and *Fasciola* antibody titer before receiving the treatment and this correlation became of a moderate significance (r = 0.4) after the treatment (Fig. 2B).

**Table 5:** Treatment of the included patients and its outcome after initial dose.

Variable	No. (261)	%
Name of the drug received		
Triclabendazole	261	100.0
Treatment response		
Single dose response	208	79.7
A second ± a third course	53	20.3
Clinical improvement		
Yes	221	84.7
No	40	15.3
Persistent symptoms or signs*: (n = 40)		
Abdominal pain	38	95
Nausea, vomiting	16	40
Fatigue	6	15
Pallor	3	7.5
Tender hepatomegaly	2	5
Itching	1	2.5
Jaundice	19	7.3
*Fasciola* eggs in stool after treatment	3	1.1
Hepatomegaly by ultrasound	13	5
Persistent hepatic focal lesion by ultrasound	25	9.6
Dilated CBD	19	7.3

*CBD* common bile duct.

*Patients may have more than one symptom or sign.

## Discussion

Fascioliasis is a major health problem, especially in limited-resource countries. In Egypt, hepatic fascioliasis was described as an endemic disease with a re-emerging pattern^24,25^. In the current study, female patients represented 55.2% compared to 44.8% of males. This is in agreement with a study by Curtale et al. that included over 21,000 children in Egypt and found that females had a significantly higher prevalence of fascioliasis than males^26^. A large study by Parkinson and others in the Bolivian Altiplano involving almost 8000 subjects found an insignificant association with sex^8^. Assiut Governorate, which is located in Upper Egypt, expressed the vast majority of our recruited cases and most of them were from rural communities, especially Manfalout District. This explains the occurrence of an outbreak of fascioliasis in this district during the study period which was reflected on the number of cases from Assiut, however this is not the true situation in Assiut or even in Egypt which is in concordance with the findings of Ramadan et al. and Hussieun et al.^27,28^. Most of the included patients were young which could be due to the exposure pattern of those who work on farms to be more liable to disease. This is in concordance with Mekky et al.^24^ who found a higher infection rate in males and in those who live in rural areas. Also, there may be a false-impression of bizarre distribution of the incidence all over the River Nile track. This could be explained by the location of the tertiary care Centers in areas with high ceiling hospitals like university institutes that work as a drainage area for such rare diseases.

In the present study, patients' age ranged from 5 to 75 years and about one-third of the included patients were students. This finding is in agreement with Parkinson and colleagues who reported that 70% of school children aged 8–11 years in the Bolivian Altiplano were affected by *Fasciola* infection^8^. Another study in agricultural communities in Peru showed that infected children had a mean age of 11 years in elementary school (10.2%) and high school (13.2%)^12^. In this study, more than two third of cases were rural residents (69.3%) because fascioliasis is a rural disease. Also, fascioliasis is considered as a zoonotic disease that tend to be presented in a high endemicity in a rural areas. This notion is obvious in our results as most of the patients (80 cases) were from the urban area explained by their origin from rural areas but resided in an urban area. Moreover, human fascioliasis is classified as plant- or food-borne fluke infections, often caused by ingestion of metacercariae attached to leaves that are eaten as vegetables^29^.

Several communities in low-income countries such as Egypt have a high prevalence of fascioliasis due to constant close contact with their livestock^29^. The problem of vegetables sold in uncontrolled urban markets is uncommon. The risks of traditional local dishes made from sylvatic plants are considered. Drinking contaminated water, beverages and juices, and washing of vegetables, fruits, tubercles and kitchen utensils with contaminated water are increasingly involved^30^.

More than 90% of cases complained of upper abdominal pain and fever. This finding was also reported in most of the published case scenarios regarding hepatic fascioliasis^31^. Hepatic focal lesions were reported in more than half of the patients and viable worm was detected in the biliary tree in 6.1% of cases before treatment. The presentation was variable due to variations in the incubation period that ranged from a few weeks to a few months with a wide range of disease severity^4,32,33^. Abdominal pain and fever are explained by the hepatic subcapsular invasion and or formation of small hepatic abscesses that occurred secondary to the immunologic reactions against the parasites^4^ and subcapsular nodule scan was detected on both sonography and CT scan^34^. Stool and blood techniques, the main tools for diagnosis in humans, have been improved for both patient and survey diagnosis^35^. Detection of *Fasciola* eggs in stool is a diagnostic test for fascioliasis^35^. In our study, the Kato Katz test was used for microscopic stool examination of eggs detection. In areas with a high prevalence and intensity of infection, the World Health Organization (WHO) suggests using the Kato Katz test, a quantitative microscopy test^36,37^. If used alone, the Kato Katz test might miss as many as one-third of infections. However, when combined with techniques that concentrate eggs, it becomes much more sensitive. Rapid sedimentation and spontaneous sedimentation are two sedimentation tests that are more sensitive than the Kato Katz^36^.

However, in this study, 5% of the examined records showed eggs in the stool before treatment. This could be owing to most patients in our study were in the acute phase and some patients did not do stool analysis for eggs (children and a few adults). This is in agreement with studies conducted by Ali et al. and Hussieun et al.^28,38^. Serodiagnosis of fascioliasis in human and animal species has been successfully carried out employing several antigenic fractions of *Fasciola*, purified antigens and recombinant antigens. Cathepsins L are the most frequently used target antigens for detecting anti-*Fasciola* antibodies^39^.

The presence of eosinophilia is a common laboratory finding to suspect parasitic infestation. In this study, there is a significant correlation between eosinophilia and *Fasciola* antibody titer before and after treatment. This is in agreement with the results of El Mekky et al. and Hussieun et al.^24,28^. All patients were treated with oral triclabendazole, and most cases responded well to a single dose of 10 mg/kg of triclabendazole. Criteria of cure include improvement of the clinical manifestations, decrease of eosinophils and anti-*F*asciola antibodies, disappearance of the hepatic focal lesions and ascites, and regression of splenomegaly.

Triclabendazole is considered the drug of choice in treating human fascioliasis^40^. One or two doses of 10 mg/kg per dose separated by 12 to 24 h are recommended by the WHO^37^. In 2019, the United States Food and Drug Administration (FDA) approved a two-dose regimen for the treatment of acute and chronic fascioliasis in people aged 6 and older^41^. The WHO endorses extensive medication administration as a method of decreasing the prevalence of fascioliasis in people living in endemic areas. Vietnam, Peru, Egypt, and Bolivia have implemented various measures to contain the infection in humans^8,12,37,42^. The prevalence of fascioliasis in Egypt dropped from 6 to 1% after the country implemented a school and community-based screening and treatment program in endemic areas^15^. Mass treatment and use of triclabendazole at irregular intervals may result in the emergence of resistant parasites^43^. Only a few cases of triclabendazole resistance in humans have been reported^44,45^. However given that triclabendazole is the only very effective medication available, reports of resistance are concerning as published by Ramadan et al.^27^.

In spite of being the first Egyptian study conducted in two centers; one in Upper Egypt and the other in Lower Egypt, to describe the situation of human fascioliasis, this study may carry some limitations. First, the sample size was relatively small and may not reflect the actual incidence of the disease. The researchers recruited cases from patients’ records with overt clinical and/or imaging findings. Second, the study was a retrospective one that carries an inhered selection bias. These defects can be solved by targeting all records in the selected governorates and follow-up of the cases after treatment on a large scale.

## Conclusion

In conclusion, human fascioliasis is not an uncommon disease, and a high index of suspicion should be raised in cases with fever, right upper abdominal pain, and peripheral eosinophilia. Further imaging workup is mandated to detect hepatic lesions. Prompt treatment by triclabendazole can serve as a standard-of-care regimen even for suspected cases.

### Supplementary Information

Supplementary Information.

## Data Availability

The datasets used and/or analyzed during the current study are available from the corresponding author on reasonable request.

## References

[1] Furst T, Duthaler U, Sripa B, Utzinger J, Keiser J (2012). Trematode infections: Liver and lung flukes. Infect. Dis. Clin. N. Am..

[2] Mas-Coma S, Valero MA, Bargues MD (2009). Chapter 2. Fasciola, lymnaeids and human fascioliasis, with a global overview on disease transmission, epidemiology, evolutionary genetics, molecular epidemiology and control. Adv. Parasitol..

[3] Cwiklinski K, O’neill S, Donnelly S, Dalton J (2016). A prospective view of animal and human Fasciolosis. Parasite Immunol..

[4] CDC (2018). Fasciola.

[5] Mas-Coma S, Agramunt VH, Valero MA (2014). Neurological and ocular fascioliasis in humans. Adv. Parasitol..

[6] Mas-Coma S, Valero MA, Bargues MD (2009). Climate change effects on trematodiases, with emphasis on zoonotic fascioliasis and schistosomiasis. Vet. Parasitol..

[7] Rosas-Hostos Infantes LR (2023). The global prevalence of human fascioliasis: A systematic review and meta-analysis. Ther. Adv. Infect. Dis..

[8] Parkinson M, O’Neill SM, Dalton JP (2007). Endemic human fasciolosis in the Bolivian Altiplano. Epidemiol. Infect..

[9] Cabada MM (2014). Fascioliasis and eosinophilia in the highlands of Cuzco, Peru and their association with water and socioeconomic factors. Am. J. Trop. Med. Hyg..

[10] Silva AEP, Freitas CDC, Dutra LV, Molento MB (2020). Correlation between climate data and land altitude for Fasciola hepatica infection in cattle in Santa Catarina, Brazil. Rev. Bras. Parasitol. Vet..

[11] Esteban JG, Flores A, Angles R, Mas-Coma S (1999). High endemicity of human fascioliasis between Lake Titicaca and La Paz valley, Bolivia. Trans. R. Soc. Trop. Med. Hyg..

[12] Cabada MM (2018). Socioeconomic factors associated with fasciola hepatica infection among children from 26 communities of the Cusco region of Peru. Am. J. Trop. Med. Hyg..

[13] Qureshi AW, Zeb A, Mansoor A, Hayat A, Mas-Coma S (2019). Fasciola hepatica infection in children actively detected in a survey in rural areas of Mardan district, Khyber Pakhtunkhawa province, northern Pakistan. Parasitol. Int..

[14] Zoghi S (2019). Human fascioliasis in nomads: A population-based serosurvey in southwest Iran. Infez Med..

[15] Caravedo MA, Cabada MM (2020). Human fascioliasis: Current epidemiological status and strategies for diagnosis, treatment, and control. Res. Rep. Trop. Med..

[16] Lotfy WM, Hillyer GV (2003). Fasciola species in Egypt. Exp. Pathol. Parasitol..

[17] Arjona R, Riancho JA, Aguado JM, Salesa R, González-Macías J (1995). Fascioliasis in developed countries: A review of classic and aberrant forms of the disease. Medicine (Baltimore).

[18] Haseeb AN, El-Shazly AM, Arafa MA, Morsy AT (2002). A review on fascioliasis in Egypt. J. Egypt. Soc. Parasitol..

[19] Periago MV (2021). Very high fascioliasis intensities in schoolchildren from Nile Delta governorates, Egypt: The old world highest burdens found in Lowlands. Pathogens.

[20] Esteban JG (2003). Hyperendemic fascioliasis associated with schistosomiasis in villages in the Nile Delta of Egypt. Am. J. Trop. Med. Hyg..

[21] Ahmad AA (2022). New perspectives for fascioliasis in upper Egypt’s new endemic region: Sociodemographic characteristics and phylogenetic analysis of Fasciola in humans, animals, and lymnaeid vectors. PLoS Negl. Trop. Dis..

[22] Adarosy HA, Gad YZ, El-Baz SA, El-Shazly AM (2013). Changing pattern of fascioliasis prevalence early in the 3rd millennium in Dakahlia Governorate, Egypt: An update. J. Egypt. Soc. Parasitol..

[23] Mas-Coma S, Bargues MD, Valero MA (2018). Human fascioliasis infection sources, their diversity, incidence factors, analytical methods and prevention measures. Parasitology.

[24] Mekky MA, Tolba M, Abdel-Malek MO, Abbas WA, Zidan M (2015). Human fascioliasis: A re-emerging disease in upper Egypt. Am. J. Trop. Med. Hyg..

[25] Hussein A-NA, Khalifa RMA (2010). Fascioliasis prevalences among animals and human in upper Egypt. J. King Saud Univ. Sci..

[26] Curtale F (2007). Human fascioliasis infection: Gender differences within school-age children from endemic areas of the Nile Delta, Egypt. Trans. R. Soc. Trop. Med. Hyg..

[27] Ramadan HK (2019). Evaluation of nitazoxanide treatment following triclabendazole failure in an outbreak of human fascioliasis in upper Egypt. PLoS Negl. Trop. Dis..

[28] Hussieun SM (2022). Studies on sociodemography, clinical, laboratory and treatment of fascioliasis patients in Assiut hospitals, Assiut governorate, Egypt. J. Egypt. Soc. Parasitol..

[29] Mas-Coma S, Valero MA, Bargues MD (2014). Fascioliasis. Adv. Exp. Med. Biol..

[30] Angles R, Buchon P, Valero MA, Bargues MD, Mas-Coma S (2022). One health action against human fascioliasis in the Bolivian Altiplano: Food, water, housing, behavioural traditions, social aspects, and livestock management linked to disease transmission and infection sources. Int. J. Environ. Res. Public Health.

[31] Gell, J.M. & Graves, P.F. Case Study: 32-Year-Old Male Presenting with Right Lower Quadrant Abdominal Pain. In *StatPearls* (StatPearls Publishing Copyright © 2023, StatPearls Publishing LLC., Treasure Island (FL) ineligible companies. Disclosure: Peter Graves declares no relevant financial relationships with ineligible companies, 2023).

[32] Lalor R (2021). Pathogenicity and virulence of the liver flukes *Fasciola* hepatica and *Fasciola*
*Gigantica* that cause the zoonosis Fasciolosis. Virulence.

[33] Kaya M, Beştaş R, Cetin S (2011). Clinical presentation and management of Fasciola hepatica infection: Single-center experience. World J. Gastroenterol..

[34] Kim TK, Jang HJ (2014). Contrast-enhanced ultrasound in the diagnosis of nodules in liver cirrhosis. World J. Gastroenterol..

[35] Mas-Coma S, Bargues MD, Valero MA (2014). Diagnosis of human fascioliasis by stool and blood techniques: Update for the present global scenario. Parasitology.

[36] Lopez M (2016). Kato-Katz and Lumbreras rapid sedimentation test to evaluate helminth prevalence in the setting of a school-based deworming program. Pathog. Glob. Health.

[37] World Health Organization (2007). Report of the WHO Informal Meeting on use of Triclabendazole in Fascioliasis Control: WHO Headquarters, Geneva, Switzerland 17–18 October 2006.

[38] Ali MN, Amin MA, Nada MS, Abou-Elez RM (2014). Fascioliasis in man and animals at sharkia province. Zagazig Vet. J..

[39] Valero MA (2012). Assessing the validity of an ELISA test for the serological diagnosis of human fascioliasis in different epidemiological situations. Trop. Med. Int. Health.

[40] Fang W, Chen F, Liu HK, Yang Q, Yang L (2014). Comparison between albendazole and triclabendazole against Fasciola gigantica in human. Zhongguo Xue Xi Chong Bing Fang Zhi Za Zhi.

[41] Zhang X (2020). Application of PBPK modeling and simulation for regulatory decision making and its impact on US prescribing information: An update on the 2018–2019 submissions to the US FDA’s office of clinical pharmacology. J. Clin. Pharmacol..

[42] Bui TD, Doanh PN, Saegerman C, Losson B (2016). Current status of fasciolosis in Vietnam: An update and perspectives. J. Helminthol..

[43] Sargison N (2012). Diagnosis of triclabendazole resistance in *Fasciola* hepatica. Vet. Rec..

[44] Winkelhagen AJ, Mank T, de Vries PJ, Soetekouw R (2012). Apparent triclabendazole-resistant human *Fasciola* hepatica infection, the Netherlands. Emerg. Infect. Dis..

[45] Gil LC (2014). Resistant human fasciolasis: Report of four patients. Rev. Med. Chil..

