# Supplementary material for: Clinical characteristics of human fascioliasis in Egypt
Source: Sci Rep. 2023 Sep 27;13:16254. doi: 10.1038/s41598-023-42957-7 (PMC10533839; doi:10.1038/s41598-023-42957-7)
Supplement: Supplementary file 1 — Supplementary Information. [file 41598_2023_42957_MOESM1_ESM.docx]

Supplementary

**Supplementary table 1:** Reported Fascioliasis cases from data records in Assiut and Al- Behera.

|  | **Assiut** | **Al- Behera** |
| --- | --- | --- |
| 2018 | 196 | 26 |
| 2019 | 112 | 20 |
| 2020 | 7 | 9 |
| **Total** | 315 | 55 |

**Supplementary Figure 1: Distribution of the studied patients by their residence**
